# The Janus Face of IL-33 Signaling in Tumor Development and Immune Escape

**DOI:** 10.3390/cancers13133281

**Published:** 2021-06-30

**Authors:** Mi-Ran Choi, Jeffrey A. Sosman, Bin Zhang

**Affiliations:** Robert H. Lurie Comprehensive Cancer Center, Department of Medicine-Division of Hematology/Oncology, Northwestern University Feinberg School of Medicine, Chicago, IL 60611, USA; mi.choi@northwestern.edu (M.-R.C.); jeffrey.sosman@nm.org (J.A.S.)

**Keywords:** IL-33, ST2, immune editing, cellular context, cancer immunotherapy

## Abstract

**Simple Summary:**

Interleukin-33 (IL-33) is often released from damaged cells, acting as a danger signal. IL-33 exerts its function by interacting with its receptor suppression of tumorigenicity 2 (ST2) that is constitutively expressed on most immune cells. Therefore, IL-33/ST2 signaling can modulate immune responses to participate actively in a variety of pathological conditions, such as cancer. Like a two-faced Janus, which faces opposite directions, IL-33/ST2 signaling may play contradictory roles on its impact on cancer progression through both immune and nonimmune cellular components. Accumulating evidence demonstrates both pro- and anti-tumorigenic properties of IL-33, depending on the complex nature of different tumor immune microenvironments. We summarize and discuss the most recent studies on the contradictory effects of IL-33 on cancer progression and treatment, with a goal to better understanding the various ways for IL-33 as a therapeutic target.

**Abstract:**

Interleukin-33 (IL-33), a member of the IL-1 cytokine family, plays a critical role in maintaining tissue homeostasis as well as pathological conditions, such as allergy, infectious disease, and cancer, by promoting type 1 and 2 immune responses. Through its specific receptor ST2, IL-33 exerts multifaceted functions through the activation of diverse intracellular signaling pathways. ST2 is expressed in different types of immune cells, including Th2 cells, Th1 cells, CD8^+^ T cells, regulatory T cells (T_reg_), cytotoxic NK cells, group 2 innate lymphoid cells (ILC2s), and myeloid cells. During cancer initiation and progression, the aberrant regulation of the IL-33/ST2 axis in the tumor microenvironment (TME) extrinsically and intrinsically mediates immune editing via modulation of both innate and adaptive immune cell components. The summarized results in this review suggest that IL-33 exerts dual-functioning, pro- as well as anti-tumorigenic effects depending on the tumor type, expression levels, cellular context, and cytokine milieu. A better understanding of the distinct roles of IL-33 in epithelial, stromal, and immune cell compartments will benefit the development of a targeting strategy for this IL-33/ST2 axis for cancer immunotherapy.

## 1. Introduction

Interleukin-33 (IL-33) was identified over a decade ago as an IL-1 cytokine family member [1] and is now recognized as a crucial player in innate and adaptive immunity. Suppression of tumorigenicity 2 (ST2), an IL-1R family member, was initially discovered as an orphan receptor and then determined to be the receptor for its ligand IL-33. The expression of IL-33 and its receptor, ST2, is found in a wide range of cells. The strongest expression of IL-33 has been observed in non-hematopoietic cells, including epithelial cells, endothelial cells, and fibroblasts. Initially, ST2 was detected exclusively by T helper 2 (Th2) cells [2] but identified later to also be expressed on the surface of other activated leukocytes, including Th1 cells, CD8^+^ T cells, and Natural Killer (NK) cells. IL-33 is one of the alarmins (also called Damage-associated molecular patterns, DAMPs), such as high-mobility group box 1 protein (HMGB1), ATP, heat-shock proteins (HSPs), and IL-1α [3,4]. In response to tissue injury, cellular stress, and infection [5], intracellular DAMPs are typically released from damaged or dying cells undergoing various forms of cell death [6], including immunogenic cell death (ICD) [4]. Despite the absence of direct experimental evidence, a potential involvement of IL-33 in ICD contributing to the control of tumor growth could be inferred from the activation of the NLR family pyrin domain containing 3 (NLRP3) in dendritic cells (DCs) [7,8]. 

**Figure 1:**
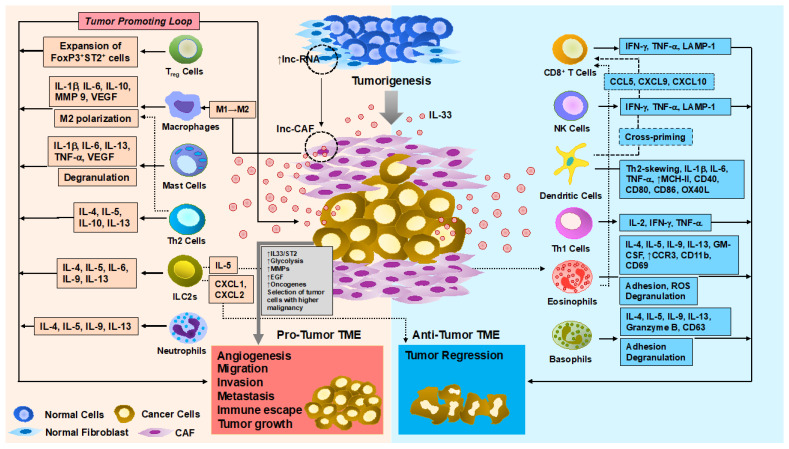
The dual role of IL-33 in regulating antitumor immune responses during tumor progression. Along with tumor development, IL-33 is likely downregulated in epithelial cells but upregulated in the TME despite the existing contradictory results. Nevertheless, the increased IL-33 expression in stromal components, such as CAF, creates highly suppressive milieu for the immune response, thus contributing to the tumor progression and metastasis mainly by recruiting immune-suppressive tumor-associated macrophages (TAM), myeloid-derived suppressor cells (MDSC), and T_reg_. By contrast, the majority of studies reveal that systemic and/or local production of IL-33 exerts a potent inhibitory effect on tumor growth and metastasis by enhancing both adaptive (i.e., effector Th1, and CD8^+^ T cells) and innate (i.e., NK cells, DCs, and eosinophils) immune components. A better understanding of the pleiotropic roles of IL-33 in the epithelial, stromal, and immune cell compartment in TME is becoming increasingly important in developing more effective approaches in cancer immunotherapeutics.

The role of IL-33/ST2 signaling has been widely investigated in various pathological conditions, including infection and allergic diseases [9,10]. In addition, this axis has also been shown to be involved in processes such as wound healing, fibrosis, and modulation of immune responses [11]. Accumulating evidence reveals that the IL-33/ST2 signaling axis is linked closely to tumorigenesis as well as to tumor immunity. Numerous studies demonstrate IL-33 plays a key role in regulating neoplastic transformation, tumor growth, and metastasis in many cancers [12]. Here we summarize the pleiotropic characteristics of IL-33 and recent advances in preclinical research with a focus on its dual role in regulating tumor immune escape (Figure 1), revealing its potential as a target for cancer immunotherapy (Table 1). 

## 2. Molecular Features of IL-33

### 2.1. Gene and Protein

A total of eight exons of the human *IL33* gene spread over 42 kb of genomic DNA [1,43], and its mRNA (2.7 kb) encodes a protein composed of 270 (266 in mouse) amino acids [1,43]. The functional activity of IL-33 is tightly regulated, which is reflected in the protein features. N-terminal- and C-terminal domains of IL-33 protein are evolutionarily conserved, and a linker region in the center is highly divergent [44]. Each domain contains a specific motif that is critical for the function of IL-33: chromatin-binding motif (45–53) in the N-terminal nuclear localization, cleavage sites for inflammatory proteases (90–112) in the central domain, and cleavage site for caspase-3 and -7 (174–179) in the C-terminal IL-1-like cytokine domain. IL-1 family members share a similarity in the C-terminal cytokine domain. Phylogenetically, IL-33 protein is conserved in mammals, and IL-18 is most related to IL-33 among the family molecules [1]. Afferni et al. analyzed large-scale cancer genomics data sets and suggested a close relationship between the development of certain cancer types and somatic mutations on the *IL33* gene [45]. Despite overall mutational frequencies of the *IL33* gene in all tumors examined remain low (0.072–1.391%), some mutations found at the specific motif in each domain of IL-33 may result in aberrant IL-33 function associated with tumor regression or progression. Systematic evaluation of the mutational effects on the biological activities of IL-33 is still to be done. 

### 2.2. Nuclear Localization and Release of Isoforms

Constitutive expression of IL-33 mRNA and protein is detected in various organs and cell types, such as epithelial cells, endothelial cells, fibroblasts, and cardiac myocytes, in human and mice [1,44,46,47,48]. Intracellular IL-33 localizes in the nucleus, associated with chromatin [44]. The histone proteins, H2A-H2B dimer forms an acidic pocket at the surface of the nucleosome, and the chromatin-binding motif in the N-terminal nuclear localization domain causes the protein to dock into the pocket [49], suggesting transcriptional repressor properties of IL-33 [44,50]. In response to endogenous and exogenous danger signals, a full-length form of intracellular IL-33 is cleaved by caspase-1 and released to extracellular space. This process requires the NLRP3 inflammasome. Danger signal-activated NLRP3 inflammasome induces caspase-1 activation, which promotes IL-33 processing and subsequent release [7]. IL-33, lacking secretory signaling peptide, is not secreted but released in its full length [44] in the extracellular space when injured cells undergo necrosis. Once released, the full-length IL-33 can be functional to trigger IL-33/ST2 signaling or cleaved at the short amino acid fragment in the central domain by inflammatory proteases. The resulting shorter forms of IL-33 (18–21 kDa) have a 10- to 30-fold higher activity than the full-length form [51,52,53]. The inflammatory proteases are released from mast cells and neutrophils which are recruited to the tumor microenvironment (TME), thereby promoting maturation of IL-33 to a higher active form within tumors. Moreover, the different effects of these forms of IL-33 can often occur. In a mouse model for lung-specific expression of IL-33 isoforms via delivery of recombinant adenovirus vectors, the full-length IL-33 promoted inflammation in an ST2-independent manner without Th2 skewing. Conversely, short mature IL-33 exerted ST2-dependent Th2-associated effects [54]. In a therapeutic mouse model for a vaccination with human papilloma virus (HPV) DNA vaccine in conjunction with either full-length or short mature IL-33 as an immunoadjuvant, both IL-33 isoforms were comparable to enhance antigen-specific effector and memory T-cell immune responses, whereas the full-length IL-33 was more potent to facilitate the humoral immune response than the mature IL-33 [55]. Alternative splice variants of human IL-33 mRNA have been identified depending on cell types and the pathological responses [56,57,58]. Three splicing variants of the IL-33 contain 7, 6, and 4 exons. Public databases, such as IsoExpresso, contain expression data of isoforms for thousands of human cancer-associated genes [59]. Analysis of these datasets revealed a differential expression of IL-33 variants throughout normal tissues and the lesions. Interestingly, breast, bladder, liver, and thyroid cancers displayed higher expression levels of variants 2 and 3 compared to normal counterparts [45]. However, the association between the expression level of the variants and their activity in pro- or anti-tumorigenic mechanisms is still ill-defined. On the other hand, intracellular IL-33 is cleaved and inactivated by activated caspases in apoptotic cells. This leads to a drastic reduction in IL-33 and eventually to the immune tolerance triggered by apoptotic cells [60,61,62]. The activity of extracellular IL-33 can also be limited by biochemical oxidation. The formation of disulfide bridges between oxidated cysteine residues in IL-33 results in conformational changes and subsequently inhibits or disrupts the binding of IL-33 to ST2 receptor, thereby rapidly inactivating IL-33 [63]. Additional regulation of IL-33 activity is dependent on sST2, a soluble isoform of ST2 that contains an extracellular domain without a transmembrane domain. sST functions as a decoy receptor to prevent interaction between IL-33 and ST2 [64,65,66,67]. Variable expression levels of sST2 accumulated in the different TME may serve as an alternative mechanism to limit IL-33 bioactivity [67]. Nevertheless, IL-33/ST2 signaling is regulated typically by a single immunoglobulin domain IL-1R-related molecule (SIGIRR) that interferes dimerization of ST2 and IL-1 receptor accessory protein (IL-1RAcP) [68].

### 2.3. Signaling Cascade

Once released into the extracellular space, IL-33 binds to ST2 on target cells via IL-1-like cytokine domain, undergoes conformational changes, thus serving as a platform to recruit a co-receptor, IL-1 receptor accessory protein (IL-1RAcP), and finally forms IL-33-heterodimer receptor complex. This, in turn, enables the activation of intracellular signaling pathways via cytoplasmic Toll/IL-1R (TIR) domains [69,70]. IL-33/ST2 signaling activates myeloid differentiation primary response 88 (MyD88)/interleukin-1 receptor (IL-1R) associated kinase (IRAK)/TNF receptor-associated factor 6 (TRAF6) pathway in the majority of cell types, including cancer cells, stromal cells, and immune cells. TRAF6 further activates mitogen-activated protein kinase kinase 7 (MAP3K7, also known as TAK1) downstream, which leads to the activation of transcriptional regulator, nuclear factor-κB (NF-κB), through signaling of stress-activated protein kinase p38, c-Jun N-terminal kinases (JNK) as well as extracellular signal-regulated kinase (ERK). 

**Figure 2:**
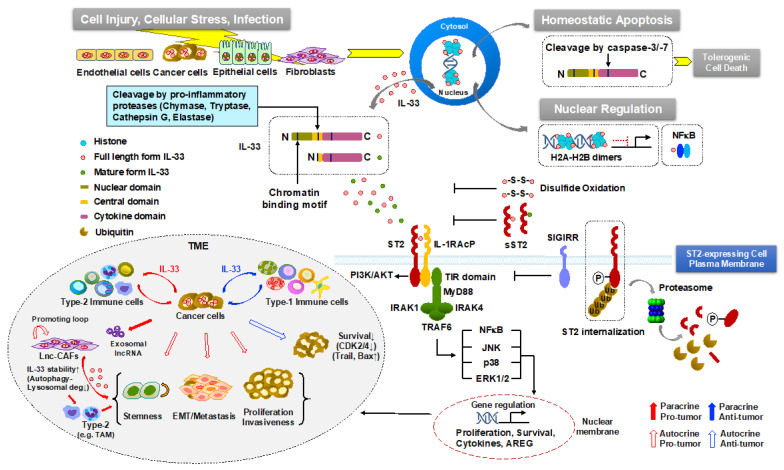
The regulation of IL-33/ST2 signaling pathway. Newly synthesized full-length IL-33 remains bound to chromatin or NF-kB in the nucleus and probably acts as a suppressive nuclear factor. Upon necrosis-inducing stimuli, such as tissue damage, environmental stress, and pathogen infection, IL-33 is released to the extracellular space. The released full-length form is either retained or cleaved to produce a mature, short-form by pro-inflammatory proteases derived from mast cells and neutrophils. The full length or mature form of IL-33 binds to its receptor ST2, allowing the dimerization with the co-receptor, IL-1RAcP. This cluster recruits intracellular adaptor molecules, MyD88, IRAK1, IRAK4, and TRAF6, leading to the activation of NF-κB and MAPKs, p38, JNK, and ERK. Notably, the IL-33/ST2 axis can be negatively regulated by several distinct mechanisms. During apoptotic cell death, IL-33 is cleaved by activated apoptotic caspases. After its release, IL-33 is blocked to bind to ST2 by oxidation at cysteine residues or by binding to sST2 that functions as a decoy receptor, therefore, abrogating IL-33/ST2 signaling. An alternative mechanism involves sequential phosphorylation of ST2, its internalization, ubiquitination followed by proteasome-mediated degradation. Additionally, the TIR domain of SIGIRR, a negative regulator, interacts with ST2 and forms a receptor complex, inhibiting the IL-33/ST2 signaling pathway. Overall, the biological consequence of IL-33/ST2 signaling on tumorigenesis and cancer treatment is complex and dictated by the site of expression, local concentration, and distribution of different IL-33 isoforms and their receptors (ST2L and sST) together with the main types of responsive cells in both autocrine and paracrine manners in the TME.

Endogenous IL-33/ST2 signaling triggers either pro- or anti-tumor immune responses across different cell types in both autocrine and paracrine manners (Figure 2). For instance, IL-33/ST2 signaling induces cancer cell stemness via the activation of JNK in human and murine colon cancer [14] or the NF-kB pathway in breast cancer [15], promoting tumor growth. On the other hand, the anti-tumorigenic effects of IL-33/ST2 signaling on inhibiting colon and pancreatic cancer cell growth were observed through autocrine regulation, by the downregulation of pro-proliferative molecules, such as CDK2, CDK4, cyclin B, cyclin D, and by the upregulation of pro-apoptotic molecules, including TRAIL, Bax, or by decreasing Flip and Bcl2 [29,30]. Furthermore, IL-33 produced by stromal cancer-associated fibroblasts (CAFs) exerts its paracrine effects on tumor invasiveness and metastasis via EMT, while distinct type-1 (e.g., cytotoxic CD8^+^ T cells, and NK cells) and type-2 immune cell components (e.g., TAM, MDSC, and T_reg_) determine their anti- and pro-tumor activities, respectively, through paracrine modulation of either cancer cell-derived or stromal IL-33 (see more details below). 

In sum, the biological consequence of IL-33/ST2 signaling on tumorigenesis and cancer treatment is complex and dictated by the site of expression, local concentration, and distribution of different IL-33 isoforms and their receptors (ST2L and sST) together with the main types of responsive cells in the TME. Pinto et al. proposed a comprehensive, integrated signaling network map of IL-33 using data mined from the published literature, which may provide more significant insights into IL-33/ST2-mediated signaling mechanism, particularly in the context of cancer [71].

## 3. Pro-Tumorigenic Function of IL-33 

The role of tumor-associated inflammation during cancer development was proposed first in the nineteenth century [72]. Numerous studies have recently demonstrated that various factors, including viral/bacterial infections [73,74], alcohol, tobacco, environmental pollutants, radiation, obesity, and high-calorie diet, are linked to the initiation and progression of cancer through chronic inflammation [75]. In particular, the implication of IL-33 acting as a “danger” signal in tumor-associated inflammation has been reported in multiple cancers, including breast cancer, gastric cancer, head and neck cancer, lung cancer, and hepatocellular carcinoma [76,77,78,79,80]. 

### 3.1. Tumor Cells

The role of the IL-33/ST2 axis has been investigated extensively in the tumorigenic processes, typically including epithelial–mesenchymal transition (EMT), migration, and invasion [11]. Wang et al. showed that the IL-33/ST2 upregulated glucose uptake and glycolysis of lung cancer cells by increasing glucose transporter I in the membrane, leading to tumor growth and metastasis in mouse model [17]. The expression level of IL-33 and ST2 in tumors has also been considered as a biomarker for patient prognosis in various cancer types. In breast cancer, the level of tumoral IL-33 and ST2 was higher than in adjacent healthy tissue. In addition, IL-33 and sST2 were elevated in serum as well, and this was correlated with poor prognosis [81]. In a mouse model for breast cancer and pulmonary metastasis, endogenous IL-33 was elevated at both mRNA and protein levels in a time-dependent manner during cancer progression [82]. The increased level of IL-33 was also observed in patients with non-small-cell lung carcinoma (NSCLC) [17] and epithelial ovarian cancer [83]. In human colorectal cancer, the levels of both mRNA and protein of IL-33 were increased in tumor cell lines, biopsies as well as the systemic circulation of patients, while genetic deficiency in IL-33 decreased tumorigenesis in an intestinal tumor model using the Apc^Min/+^ mice [25].

Accumulating evidence documents that IL-33/ST2 axis functions as a dynamic modulator of the TME. An early study examining IL-33 in tumorigenesis in mouse mammary carcinoma showed that the deletion of ST2 in BALB/c mice resulted in a decrease in tumor growth and metastasis [26], and this was concomitant with increased levels of cytokines, such as interferon (IFN)-γ, tumor necrosis factor (TNF)-α, IL-17, and decreased IL-4 in serum. Modulation of the levels of IL-33 in breast cancer by exogenously administered IL-33 resulted in the promotion of tumor growth and metastasis [27]. During intestinal inflammation and carcinogenesis, IL-33 production and ST2 receptor expression were increased [84]. In intestinal epithelial cells, the level of IL-33 was regulated by epidermal growth factor (EGF) as a key factor, and the inhibition of the EGF receptor led to the reduced level of IL-33 transcripts in vivo. The IL-33/ST2 axis is also involved in promoting tumor cell proliferation and colony formation in a COX2/PGE_2_-dependant fashion in colorectal cancer [85]. In an in vitro study, IL-33 was able to directly induce migration and invasion of the A549 lung cancer cells [18]. It was suggested that IL-33 may also indirectly promote proliferation, the transformation of tumor cells, and metastasis through cooperation with oncogenes [19,86,87]. Akimoto et al. reported that sST2 was downregulated in high-metastatic cells but not in low-metastatic colorectal cancer cells in human and mouse [13]. Being a negative regulator, sST2 could inhibit tumor growth and metastasis by suppressing IL-33-induced angiogenesis, Th1-, Th2-immune responses, as well as the infiltration and M2 polarization of macrophages. On the other hand, an unexpected role of the IL-33 receptor, ST2L (a transmembrane isoform), was also implicated in metastatic lung cancer [20]. Only low-metastatic but not high-metastatic cells derived from lung cancer tissues expressed functional ST2L. Consequently, IL-33 released from the TME induced oncosis (a nonprogrammed or accidental type of cell death characterized by swelling) [88] only in ST2L-positive low-metastatic cells in the nutrient-deprivation and hypoxia regions. In this regard, IL-33 provided selective pressure for the ST2L-negative, oncosis-resistant high-metastatic cells under conditions mirroring the TME. These data suggest an indirect (passive) role of the IL-33/ST2L axis in promoting lung cancer by selecting for more malignant cells in the TME. In addition, the contribution of IL-33 to inducing immunosuppressive TME, thus resulting in the conditions in favor of tumorigenesis, has been reported in in vitro and in vivo studies on many cancer types, including colorectal cancer, cervical cancer, gastric cancer, head and neck squamous cell carcinoma (HNSCC), lung cancer, pancreatic cancers [89]. 

### 3.2. Stromal Cells

Cancer-associated fibroblasts (CAFs) are the major non-immune cells capable of promoting tumorigenesis in the TME. IL-33-producing CAFs have been shown to induce invasiveness in HNSCC [22]. In this study, IL-33 promoted migration and invasion of the cancer cells through the induction of EMT, and the levels of IL-33 in CAFs and cancer cells were correlated with the severity of invasiveness of the HNSCC. In oral squamous cell carcinoma, a long non-coding (lnc)-RNA upregulated in CAFs was identified to interact with IL-33, which enabled the elevated expression of CAF markers and converted the normal fibroblast to CAFs. The pro-tumorigenic effect of CAFs with upregulated lnc-RNA was achieved by promoting IL-33 stability [23]. Moreover, genetic deletion of IL-33, ST2, or MMP-9 evidently inhibited metastasis in fibroblast-rich human and mouse pancreatic cancers [24]. The metastasis-promoting effect of IL-33 expressed in CAFs was associated with the switch of tumor-associated macrophages (TAMs) from anti-tumorigenic M1 to pro-tumorigenic M2 phenotype. In another study, IL-33 stimulated the proliferation and invasiveness of decidual stromal cells through MMP2 upregulation, and this could be abolished by the administration of exogenous sST2 [90]. In mouse models of spontaneous breast cancer metastasis, upregulation of IL-33 in metastases-associated fibroblasts instigated type 2 inflammation in the metastatic microenvironment and mediated recruitment of eosinophils, neutrophils, and inflammatory monocytes to lung metastases, further indicating a pivotal role of IL-33 expressing CAF in establishing a hospitable inflammatory niche in lung metastases [91]. These studies together implicate that the stromal cells in the tumor microenvironment are not just a bystander but provide IL-33 to the TME in a paracrine manner, facilitating tumor progression primarily by autocrine expression of IL-33. 

### 3.3. Immune Cells 

IL-33 is well known for its stimulating role of both myeloid and lymphoid cells and their production of type 2 cytokines [64,92]. ST2-expressing (ST2^+^) lymphoid cells include Th2, T_reg_, and ILC2 cells. ST2 in Th2 cells is expressed in a GATA3-dependent manner and mediates Th2 effector functions [2,93]. In CD4^+^ T cells, ST2 expression is mediated by IL-2-mediated STAT5 signaling [94], and in ILC2 cells, ST2 activation leads to the production of type 2 cytokines, IL-5, and IL-13 [95,96,97,98,99,100,101,102,103]. IL-33 stimulates ST2^+^T_reg_ cells and AREG expression, which enhances immune regulatory functions and tissue repair [104,105]. ILC2 activation through IL-33 supports type 2 immune responses as well as tissue repair through AREG production and the generation of reparative M2 macrophages. ST2 is also expressed in myeloid-derived antigen-presenting cells, such as macrophages and CD11b^+^CD11c^+^ dendritic cells (DCs) [106,107,108,109]. In addition, IL-2 released from IL-33-activated mast cells [110] and murine DCs [106] mediates the expansion of the T_reg_ cell population. The type, density, and location of these immune cells modulated by IL-33 within tumors influence the behavior of tumors, which are eventually associated with clinical outcomes [111,112]. 

#### 3.3.1. CD4^+^ T_reg_ Cells

ST2 is expressed on regulatory T cells (T_regs_) constitutively, depending on tissue and disease types. The IL-33/ST2 axis can recruit T_regs_ in the TME and activate their immunosuppressive function, supporting tumor immune escape, favoring tumor growth [113,114,115,116]. In a study of collagen-induced arthritis (CIA), IL-33 induced the expansion of eosinophils, Th2 cells, and ILC2s, especially the T_reg_ population. These T_reg_ cells revealed enhanced abilities to suppress IFN-γ production by effector T cells, suggesting IL-33 is capable of promoting the immunosuppressive properties of T_reg_ cells [117]. In Apc^Min/+^ mice, IL-33 derived from epithelial cells enhanced the proliferation of ST2^+^ T_reg_ cells and induced Th2 cytokine milieu in the gut, which was correlated with increased tumor burden [28]. Similarly, in the CT26 adenocarcinoma mouse model, administration of recombinant IL-33 (rIL-33) enhanced, while inhibition of IL-33 diminished, the expansion of ST2^+^ T_reg_ cells in tumor tissue and spleen, resulting in tumor growth [118]. In esophageal squamous cell carcinoma (ESCC), IL-33 stimulated the production of CCL2 through TGF-β involving T_reg_ cell recruitment [119]. Another study with a mouse lung adenocarcinoma model showed that deletion of T_reg_-specific ST2 enhanced tumor-infiltrating CD8^+^ T cells and reduced tumor burden, suggesting a critical function of ST2 in T_reg_-mediated immunosuppression in cancer [114]. In a study for sporadic colon cancer using reciprocal bone marrow chimeras in mice, the genetic deletion of ST2 in hematopoietic and non-hematopoietic compartments led to enhanced infiltration of ST2^+^Foxp3^+^ T_regs_ into tumors and increased colon tumorigenesis [31]. ST2^+^ T_reg_ expansion was shown to be amplified in a feedback loop, in which IL-33-stimulated mouse CD11c^+^ DCs were capable of secreting IL-2 to expand ST2^+^-CD4^+^Foxp3^+^ T_regs_ selectively [106]. Hatzioannou et al. demonstrated the involvement of IL-33 in the functional stability of T_reg_ cells. In their in vivo study, suppressive properties of T_reg_ cells were attenuated in *IL33*^−/−^ T_reg_ cells, facilitating tumor regression in a ST2-independent manner. These activated cells displayed epigenetic re-programming allowing elevated chromatin accessibility of the *IFNγ* locus, thus leading to increased IFN-γ production in a NF-κB/T-bet-dependent fashion [115]. In addition, Son et al. revealed upregulation of the genes for proliferation, immune suppression, and cytokine/chemokine receptor in tumor-infiltrating T_regs_ in comparison to tumor-infiltrating CD4^+^Foxp3^−^ conventional T cells or splenic T_regs_ from the same tumor-bearing mouse, confirming IL-33/ST2 axis as a critical pathway for the preferential accumulation of T_regs_ in the TME [116]. Overall, these observations provide evidences that T_regs_ activated by IL-33/ST2 signaling are enriched in TME and exert pro-tumorigenic function in contrast to their counterpart CD4^+^ T cells. 

#### 3.3.2. Macrophages

Several lines of evidence demonstrate that IL-33/ST2 signaling in macrophages is involved in M2 polarization, immunosuppression, thus promoting tumor progression. In a mouse xenograft model for human NSCLC, IL-33 mediated the amplification of the M2 markers on macrophages in vitro and in vivo, thereby facilitating the suppressive activity of TAMs. In contrast, blockade of IL-33 revoked the polarization of TAMs into M2 macrophages [120]. In addition, in mouse models of breast [27] and colon cancer [13,14,28,121], IL-33 induced the recruitment of M2-like macrophages into the TME, contributing to tumor progression. M2-like TAMs stimulated by IL-33 produced prostaglandin E_2_ (PGE_2_) and thereby facilitated colon cancer stemness and tumor growth [14]. Furthermore, IL-33-mediated recruitment of M2-like TAMs promoted tumor metastasis [122]. IL-33 directly induced MMP-9 expression in the murine macrophage cell line RAW264.7 [123] that could possibly be linked to tumor invasion and angiogenesis. Additionally, a recent study using a murine model of squamous cell carcinoma reported that tumor-initiating cells (TICs) promoted the release of IL-33 and facilitated the differentiation of macrophages expressing FcεRIα^+^ that in turn exerted paracrine TGF-β signals to TICs with inducible invasive and drug-resistant properties, while further upregulating IL-33 [124]. 

#### 3.3.3. Myeloid-Derived Suppressor Cells (MDSCs)

MDSCs, different from granulocytes and monocytes, expand in pathological (such as cancer) but not in healthy conditions and play a critical role in immune suppression [125]. A number of studies with various tumor models reported that IL-33 is capable of expanding MDSCs during cancer progression. Administration of exogenous IL-33 in 4T1 breast tumor-bearing mice enhanced systemic and intratumoral accumulation of CD11b^+^Gr-1^+^MDSCs with a higher frequency of monocytic MDSCs compared to their granulocytic subset [27]. Moreover, IL-33 promoted their immunosuppressive activity of MDSCs by upregulating the expression and activity of arginase-1 as well as activating MAPK/NF-κB signaling [126]. In addition, the accumulation, proliferation, as well as immunosuppressive activity of MDSCs were diminished in tumor-bearing mice in the absence of IL-33/ST2 signaling [27,126]. On the other hand, several conflicting evidences showed that IL-33-treated BM-derived MDSCs exhibited a reduced ability to inhibit T-cell proliferation and IFN-γ production and to produce ROS [127]. In the mouse B16 melanoma model, administration of IL-33 decreased accumulation of MDSCs in the spleen and TME [37,127], suggesting IL-33 may counteract MDSCs expansion and function relying on the tumor type. Despite few contradictory observations, the expansion and immunosuppressive role of MDSCs were augmented in a context-specific fashion by IL-33/ST2 signaling.

#### 3.3.4. Neutrophils

The roles of IL-33 in neutrophil-associated tumor immunity are ill-defined. Depending on the context of pathological conditions, IL-33 may induce inflammatory or regulatory neutrophils. The gene expression profile in IL-33-stimulated neutrophils was distinctive from that in resting or LPS-treated neutrophils. In particular, IL-33 promoted the expression of Th2 cytokines, such as IL-4, IL-9, and IL-13, in murine neutrophils in a time- and dose-dependent fashion compared to LPS-induced neutrophils [128]. In a murine model for choriomeningitis virus-induced hepatitis, IL-33 increased recruitment of neutrophils in the liver, inhibiting T-cell activation and eventually decreasing liver injury. Liver neutrophils revealed an immunosuppressive phenotype as characterized by increased levels of arginase-1, IL-10, and iNOS [129]. In the mouse CT26 colon carcinoma model, systemic treatment of mice with Irinotecan induced intestinal mucositis in association with inducible IL-33 expression and enhanced neutrophil accumulation in the intestine. Furthermore, blocking IL-33 decreased mucositis and extended treatment of ectopic colon carcinoma with Irinotecan, resulting in a favorable outcome of the chemotherapy. These data suggest that intervention of the IL-33/ST2 signaling may be beneficial for limiting mucositis and improving chemotherapy [130]. 

#### 3.3.5. Mast Cells

Mast cell infiltration is observed in various types of human and mouse tumors [131,132]. IL-33 activates human mast cells through its receptor complex (ST2 and IL-1RAcP) [133] to release cytokines by cooperation with IgE- and non-IgE-dependent stimuli [134]. Likewise, IL-33 enhanced the production of substance P-mediated vascular endothelial growth factor (VEGF) from human mast cell lines [135], indicating the potential role of IL-33-stimulated mast cells in chronic inflammation and tumorigenesis [136]. The involvement of IL-33-mediated mast cell response has been observed in various pathological conditions. In a mouse model for UV-induced skin cancer, mast cells have been found to accumulate together with fibroblasts expressing IL-33 that may evade immune destruction [137]. In the *Apc^Min^*^/+^ mouse model, IL-33 deficiency decreased the tumor burden [25,138], likely due to, in part, lowered mast cell frequency in polyps and diminished expression of mast cell-derived proteases and cytokines that may facilitate the angiogenesis, MDSC recruitment, and suppressive function of T_reg_ cells [139,140,141]. Nevertheless, it remains arguable about the pro-tumoral activity of mast cells because of the diverse range of mediators they release [142].

#### 3.3.6. ILC2

ILCs share characteristics of their cell morphology with classic lymphoid cells. ILCs have been divided into three distinct subgroups (ILC1, ILC2, and ILC3) based on profiles of effector cytokines, production of transcription factors, and phenotypic markers [102]. IL-33 preferentially acts on ILC2s and drives their expansion and recruitment [143,144,145,146]. The activated ILC2s produce cytokines including IL-4, IL-5, IL-6, IL-9, and IL-13 [95], further promoting Th2 differentiation [147]. The increased frequency of ILC2s was observed in peripheral blood of gastric cancer patients, which was potentially linked with the upregulation of Th2, MDSCs, and M2 macrophages that promote tumorigenesis [148]. ILC2s might also impair antitumor immune responses by secretion of amphiregulin, which increases the suppressive activity of T_regs_ [96,149]. In mouse tumor models, particularly in the absence of an adaptive immune system, we reported that IL-33 promoted the expansion of active ILC2s via ST2 that facilitated tumor growth by diminishing NK cell activation and tumor-killing [150]. One more recent study reported the presence of ILC2 in human pancreatic ductal adenocarcinomas (PDACs) [151]. Importantly, endogenous IL-33/ST2 signaling boosted tissue-specific cancer immunity by activating tumor-infiltrating ILC2s to prime CD8^+^ T cells in mouse models of PDAC [151]. On the other hand, in a mouse model for metastatic lung tumor, systemic administration of IL-33 induced the expansion of IL-5-producing ILC2s, which recruited and sustained eosinophils, critically contributing to increased tumor cell death, and thus preventing metastasis [152]. The IL-33/ILC2/eosinophil axis-mediated antitumor effect was also reported in association with levels of lactate produced by melanoma cells [153]. Local production of IL-33 induced intratumoral accumulation of ILC2s in mice and stimulated ILC2s to secrete CXCR2 ligands that further caused tumor cell-specific apoptosis [38]. An additional study showed that IL-33 dependent tumor-infiltrating ILC2s were able to mediate tumor immune surveillance by cooperating with DC to promote cytolytic CD8^+^ T cell responses [32]. Taken together, these findings suggest the divergent effects of IL-33/ST2/ILC2 on tumor immunity in different tissues through multiple distinct mechanisms. 

## 4. Anti-Tumorigenic Function of IL-33

### 4.1. Tumor Cells

Alongside pro-tumorigenic effects attributed to the IL-33/ST2 axis, many studies provided evidences implicating an opposite role of IL-33 in tumorigenesis. They reported that a decreased level of tumoral IL-33 and ST2 was correlated with enhanced tumor burden. Lower levels of protein and mRNA of IL-33 were found in severe cervical intraepithelial neoplasia (CIN) tissues compared to cervical tissues of patients with mild or no CIN [33]. Patients with colorectal adenocarcinoma [154], multiple myeloma [155], breast cancer [79], and lung cancer [156] also showed a negative correlation of IL-33 expression levels with tumor stage. O’Donnell et al. reported reduced expression of ST2L in human colorectal cancer tissue compared to adjacent non-tumor tissue, associating with poorer prognosis [121]. Moreover, the expression of IL-33 was decreased in multiple carcinomas during metastatic progression [34]. Parallel studies in human indicated low intratumoral IL-33 as an immune biomarker for recurrent prostate and kidney renal clear cell carcinomas. Importantly, in pancreatic cancer, the anti-tumorigenic activity of IL-33 has been demonstrated through tumor cell-intrinsic downregulation of cellular proliferating proteins, such as CDK2 and CDK4, and upregulation of pro-apoptotic molecules, such as Bax and TRAIL [29]. 

### 4.2. Immune Cells

The anti-tumorigenic activity of IL-33 is highlighted in its ability to trigger type 1 immune responses. The key players of type 1 immune response include CTL, NK cells, NKT cells, IFN-γ-producing Th1 cells, and γδ T cells [111,112]. Expression of ST2 on these immune cells implicates the involvement of IL-33/ST2 in innate and adaptive type 1 immune response [157,158,159,160]. Critical regulators for the effective action of the adaptive anti-tumor immunity, such as T-bet and Eomes, have been identified in tumor-infiltrating CD8^+^ T cells and Th1 cells [112]. The type 1 immune response activated by the IL-33/ST2 axis is characterized by enhanced production of cytokines, such as IL-2, IL-12, IFN-γ, and TNF-α [35,159,161,162,163]. 

#### 4.2.1. CD4^+^ Th Cells

Naïve CD4^+^ Th cells express ST2 constitutively. Stimulation with IL-33 preferentially skews the cell differentiation towards the Th2 phenotype, which is traditionally considered to favor tumor growth. However, recent studies demonstrated that IL-33 can induce an anti-tumor response via Th1 activity. Indeed, IL-33, as an effective adjuvant in combination with an HPV16 E6/E7-encoded DNA vaccine, exerted anti-tumorigenic function through increased production of IFN-γ^+^ and TNF-α by antigen-specific CD4^+^ T cells, and through increasing the concentration of antigen-specific serum IgG, resulting in tumor regression in mice [55]. Similarly, endogenous IL-33 promoted the expansion of IFN-γ^+^CD4^+^ T cells in tumor mouse models for colon carcinoma [164,165] and hepatocellular carcinoma [166]. It has also been shown that IL-33-mediated CD4^+^ Th1 differentiation depends on IL-12. IL-33, together with IL-12, enhanced the expression of their receptors ST2 and IL-12R during the early activation of CD4^+^ T cells. These results suggest that IL-33 preferentially facilitates Th1 cell development but not mature Th1 cells [167]. Although the precise mechanism for IL-33-induced Th1 polarization remains elusive, it is proposed that IL-33-mediated Th2 or Th1 response relies on the cytokine milieu, especially on the balance of IL-4 and IL-12 levels [167]. IL-12 signaling stimulates not only Th1 cell development and IFN-γ production by CD8^+^ T cells but also ST2 expression on type 1 immune cells, including Th1 cells, CD8^+^ T cells, and NK cells [35,158,159,161,162,163], thereby enhancing the ability of these cells to produce IFN-γ upon IL-33 exposure [157,158,162,168]. In addition, IL-33 can facilitate the differentiation of IL-9 producing Th cells (Th9), conferring their anti-tumor response in melanoma [169]. These data indicate a distinct mechanism for IL-33 to redirect the type 2 immune response to alternative differential effector functions [64,161]. 

#### 4.2.2. CD8^+^ T Cells

The expression of ST2 has been observed in effector CD8^+^ T cells and polarized Tc1 cells [159]. High expression levels of ST2 were achieved in Tc1 cells by T-bet. IL-33, together with IL-12, was able to induce IFN-γ release in Tc1 cells [159] synergistically. Gao et al. showed that tumor growth, as well as metastasis, were attenuated in mouse models for several tumors overexpressing IL-33, and this effect was accompanied by enhanced proliferation and infiltration of activated CD8^+^ T cells and cytotoxic NK cells [35]. In a mouse model for hepatocellular carcinoma, ectopic expression of IL-33 in tumor cells or hydrodynamical injection of IL-33–expressing plasmids into the liver of tumor-bearing mice inhibited tumor development by promoting effector CD4^+^ and CD8^+^ T cell activation and IFN-γ production [166]. In an aggressive mouse AML model, we demonstrated that rIL-33 treatment enhanced tumor protective effects by strongly increased expansion of leukemia-reactive CD8^+^ IFN-γ^+^ T cells, which resulted in delayed leukemia development and increased overall survival. We also showed the administration of rIL-33 suppressed the growth of established melanoma tumors by increased expansion, tumor infiltration, and improved effector function of antigen-specific CD8^+^ IFN-γ^+^ T cells directly and indirectly through activation of myeloid dendritic cells (mDCs) [40]. In addition, endogenous IL-33 production is also responsible for an effective anti-tumor response through CD8^+^ T cells. In mouse models of colon carcinoma, endogenous IL-33 overcame the pro-tumorigenic effect of T_reg_ by increasing IFN-γ production of tumor-infiltrating CD4^+^ and CD8^+^ T cells, as well as the accumulation of these infiltrating CD8^+^ T cells, exerting their anti-tumor effect [164]. Consistent with these results, the blockade of ST2 has been shown to diminish infiltration of CD8^+^ T cells into the tumors and promote tumor growth [121] in a similar mouse model of colon cancer [121].

#### 4.2.3. NK and NKT Cells

The IL-33/ST2 axis is involved in the expansion of NK cells and promotes host defense during viral infection [170,171]. Likewise, IL-33-activated NK cells producing IFN-γ contribute to anti-tumor immunity by direct killing of tumor cells. IL-33, along with IL-12, has been shown to activate NK and NKT cells in both human [158] and mouse [157], inducing IFN-γ production, and thus enhancing Th1 immunity. Transgenic expression of IL-33 in metastasis models for B16 melanoma and Lewis lung carcinoma resulted in increased recruitment of cytotoxic NK cells to the lung site that limited metastatic progression [41]. Furthermore, overexpression of IL-33 in B16 and 4T1 tumor cells inhibited tumor growth, correlating to increased tumor infiltration of IFN-γ^+^ NK cells [35]. Consistent with these results, the administration of exogenous IL-33 augmented frequencies of CD107a^+^IFN-γ^+^ NK cells in tumors and spleens of B16 melanoma-bearing mice [37]. A more recent study further reported the potent inhibition of pulmonary metastasis of murine breast cancer by IL-33 administration, which was associated with the elevation of recruited NK cells to the TME [172]. Conversely, the Lukic group found that in ST2-deficient mice bearing 4T1 breast tumors, the number of activated NK cells and their cytotoxicity were increased compared to ST2-proficient mice. Furthermore, depletion of NK cells in vivo in ST2-deficient mice led to expedited tumor growth [26]. Moreover, the administration of exogenous IL-33 to ST2-proficient tumor-bearing mice reduced activation and cytotoxic activity of NK cells, promoting tumor growth [27]. The conflicting results regarding the role of IL-33 in modulating NK cell activation and recruitment warrant extensive investigations.

#### 4.2.4. DCs

Despite low expression levels of ST2 on DCs, IL-33 was capable of activating these cells by upregulating MHCI-II and costimulatory molecules, such as CD40, CD86, and CD80, as well as a variety of cytokines/chemokines, including IL-4, IL-5, IL-1β, CCL17, and CCL22 [108,173,174,175]. IL-33 expanded BM-derived DCs by inducing GM-CSF secreted from basophils [176]. In addition, DCs, upon activation by IL-33, elicited an atypical Th2 type of immune response, activating CD4^+^ T cells to produce IL-5 and IL-13 in vitro and in vivo [108,174], and further amplifying allergic inflammatory responses via ST2 [173,177]. In our study on a murine AML model, we showed that systemic administration of IL-33 induced DC activation and “licensing” for cross-priming of tumor-reactive CD8^+^ T cells [39]. Similarly, in our melanoma models, exogenously administered IL-33 activated mDCs in the TME, promoting cross-presentation of tumor antigen in a ST2/MyD88/STAT1-dependent fashion and restoring a competent anti-tumor T cell activity [40]. The importance of the IL-33/ST2/MyD88 axis for tumor inhibition was further confirmed in the CD8^+^ T cells-mediated tumor immunity through mDCs in a mouse model of Lewis lung carcinoma [178]. These data demonstrated that IL-33 can boost tumor-associated DC functionality, thereby contributing to anti-tumor T cell immune responses. 

#### 4.2.5. Eosinophils

Eosinophils have been found to infiltrate into many types of tumors and play divergent roles in tumor development [179]. In general, eosinophils migrate to the TME in response to eotaxin-1, -2, and -3 (CCL11, CCL24, and CCL26), which bind to the receptor (CXCR3) expressed on eosinophils [179,180] or to the alarmin signals, such as IL-33 and HMGB1 [37]. In contrast to HMGB1, which attracts eosinophils directly, IL-33-mediated recruitment of eosinophils seems to require indirect attractants, including activated IL-5-producing ILC2 [152,153,181], mast cells [131], or stimulation of chemokines released from tumors, such as CCL24 [182,183]. On the other hand, administration of rIL-33 has been shown to cause massive infiltration of eosinophils and elevated levels of typical type 2 cytokines (e.g., IL-5, IL-9, and IL-13), contributing to fibrosis and allergy in mice [184]. IL-33 also maintained the ST2-dependent survival of eosinophils by autocrine GM-CSF [185]. A number of studies demonstrated the crucial role of eosinophils in the IL-33-mediated antitumor effect. IL-33 treatment [37] and its tumoral expression [186] in a mouse model for melanoma restricted tumor growth, which was abrogated by the depletion of eosinophils using monoclonal antibodies. In another melanoma model, IL-33 induced proliferation of innate IL-5-producing non-T cells upon tumor invasion, and the regulation of eosinophils by these IL-5-producing cells appeared to be critical to limit tumor metastasis [152]. Likewise, Lucarini et al. reported in another B16 melanoma model that IL-33 inhibited primary tumor growth and lung metastasis by recruiting and activating eosinophils. The anti-tumor effects of IL-33 were completely abrogated by the depletion of eosinophils. In addition, IL-33-activated eosinophils gained cytotoxic functions against melanoma cells in vitro [37]. IL-33-activated eosinophils in the TME were capable of restricting tumor growth in mouse models of colorectal cancer [183]. This was abrogated in eosinophil-deficient ΔdblGATA-1 mice, but the adoptive transfer of eosinophils activated ex vivo restored the inhibition of tumor growth. Further studies revealed that eosinophils contribute to anti-cancer immunity by supporting the recruitment of tumor-reactive CD8^+^ T cells and/or by a direct cytotoxic effect of eosinophils [37,182,183,187]. As a matter of fact, eosinophils attracted CD8^+^ T cells to TME through secreting chemokines, such as CCL5, CXCL9, and CXCL10 [187]. In addition, IL-33 induced the tumoricidal effect of eosinophils, directly killing tumor cells by increasing the effector molecules, such as markers of degranulation (CD63, CD107a), activation (CD69), and adhesion molecules, such as CD11b/CD18 and ICAM-1 [182,183,188,189]. Once activated by IL-33, eosinophils adhered to cancer cells via CD11b/CD18 and degranulated lytic molecules through their convergence to immunological synapses [182].

#### 4.2.6. Basophils

Human basophils express ST2 constitutively that can be further induced by IL-33 [190,191]. IL-33, exerting priming effects, enhanced the degranulation of human basophils and IL-4 release in response to IgE cross-linking [190]. IL-33 can activate human basophils and enhance IL-3- and anti-IgE-induced degranulation of basophils, cytokine production, and histamine secretion [190,192]. In human basophils, IL-33, in synergy with IL-3, stimulates IL-9 production [193], which may strengthen tumor immunity [194]. In mouse melanoma models, tumor-infiltrating basophils seemed to increase infiltration of CD8^+^ T cells by producing chemokines CCL3 and CCL4, leading to tumor rejection after depletion of T_reg_ cells [195]. Clinically, poor prognosis in CRC patients has been shown to be associated with low numbers of circulating eosinophils and basophils [196]. Recently, Marone et al. reported that IL-33-stimulated basophils increased the expression of degranulation marker (CD63) and of granzyme B, resulting in the killing of melanoma tumor cells in vitro [197]. Although the role of IL-33 in regulating basophils in anti-cancer immunity is poorly characterized [198], these findings may widen the scope of effector immune cells in TME activated by IL-33. 

## 5. Targeting IL-33/ST2 to Augment Cancer Immunotherapy

A number of the aforementioned studies have demonstrated a potent antitumor effect of systemic administration of rIL-33 in multiple mouse tumor models. Interestingly, we showed the inducible expression of immune checkpoint molecule PD-1 on effector CD8^+^ T cells and elevated levels of PD-L1 on tumor cells by IL-33 treatment, thereby providing a strong rationale for combination therapy of IL-33 and immune checkpoint blockade (ICB). Indeed, a combination of IL-33 treatment with anti-PD-1 resulted in improved survival of leukemic mice compared to each therapy alone. This was the first proof-of-concept evidence of synergistic therapeutic efficacy between IL-33 treatment and ICB. In a mouse model for breast cancer, administration of exogenous IL-33 increased NKp461^+^ PD-1^+^ cell population, but not in ST2-deficient mice [27]. Another study using a B16.OVA melanoma model revealed that systemically delivered IL-33 in combination with dectin-1-activated BM-derived DCs promoted PD-1 expression in OVA-specific CD4^+^ T cells, suggesting modulation of PD-1 levels in T cells via DC stimulation [199]. Several studies reported that IL-33 was capable of enhancing the expression of PD-1 on ILC2 cells, leading to impaired production of Th2-type cytokines [200,201]. Interestingly, in a PDAC mouse model, IL-33 upregulated PD-1 specifically on Tumor ILC2 but not on draining LN ILC2. IL-33 treatment in combination with PD-1 blockade reduced tumor growth in an ILC2-dependent manner, indicating that Tumor ILC2 was directly targeted by anti-PD-1 [151]. These findings suggest a modulatory function of IL-33 on PD-1/PD-L1 signaling in multiple cell types, enabling a superior efficacy of combination therapy of IL-33 plus ICB. A recent study showed an additional function of IL-33 in ICB. After treatment with anti-PD-1 and anti-CTL antigen-4 (CTLA-4) monoclonal antibodies (mAbs), IL-33 induced in tumor cells led to the improved anti-tumor efficacy of checkpoint inhibitors by increasing the accumulation and effector function of tumor-resident CD103^+^CD8^+^ T cells and the numbers of CD103^+^ DC in the TME. The combination of IL-33 with anti-PD-1 and anti-CTLA-4 antibodies further prolonged the survival of tumor-bearing mice [202]. CTLA-4 is expressed on regulatory T cells and serves as a suppressive signal of T cell response [203]. Little is known about how to modulate CTLA-4 by IL-33. In a pulmonary metastasis model derived from murine melanoma, IL-33 enhanced the frequency of CD8^+^ T cells expressing CTLA-4, PD-1, and KLRG-1 [204]. An increasing number of studies have reported a positive correlation between local IL-33, eosinophils in the TME, and improved efficacy of anti-CTLA-4/anti-PD-1 immunotherapy [186,205,206]. 

**Figure 3:**
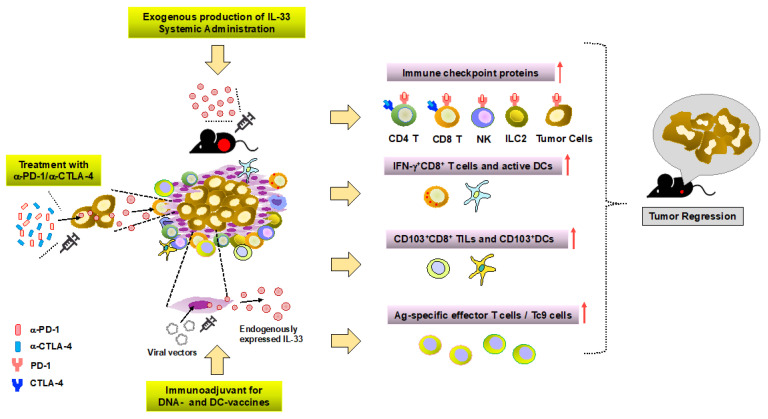
Targeting IL-33/ST2 to augment cancer immunotherapy. IL-33 upregulates PD-1 and/or PD-L1 on tumor cells and different immune cells, such as effector CD4^+^CD8^+^, NK, and ILC2 cells, thereby enabling a superior efficacy of combination therapy of IL-33 plus immune checkpoint blockade (ICB). In addition to ICB, IL-33-mediated cancer vaccine has been implicated as an additional option for immunotherapy. Moreover, the engineered vector targeted IL-33 expressing cells provides novel means to trigger the IL-33/ST2 signaling and subsequently effector T cell-mediated antitumor immune responses. IL-33 treatment also drove the induction and cytotoxic activities of DC-induced Tc9 cells, a subset of effector CD8^+^ T cells producing cytotoxic IL-9, and further promoted the therapeutic efficacy of DC-based tumor vaccines. Notably, the intervention of the IL-33/ST2 axis for boosting cancer immunotherapy should be approached with caution, given that IL-33 is a pleiotropic cytokine with the nature of a double-edged sword, namely pro- and anti-tumorigenic functions.

In addition to ICB, the IL-33-mediated cancer vaccine has been implicated as an additional option for immunotherapy. Villarreal et al. showed that IL-33 constructs served as a potent immunoadjuvant in a HPV DNA cancer vaccine platform [55]. Besides the direct delivery of IL-33, an interesting study reported endogenous IL-33 was released from stromal cells targeted by a lymphocytic choriomeningitis virus (LCMV)-based replication-competent vector delivering tumor-specific antigen to DC for efficient priming. This engineered vector targeted IL-33 expressing cells provides a novel means to trigger the IL-33/ST2 signaling and subsequently effector T cell-mediated antitumor immune responses [207]. IL-33 treatment also drove the induction and cytotoxic activities of DC-induced Tc9 cells, a subset of effector CD8^+^ T cells producing cytotoxic IL-9, and further promoted the therapeutic efficacy of DC-based tumor vaccines [208]. Apart from triggering the IL-33/ST2 signaling to augment cancer immunotherapy, a number of studies showed the blockade of IL-33, ST2, or both resulted in growth inhibition of tumors [120,209,210] in association with reduced accumulation of ST2^+^ tumor-promoting cells, such as T_reg_, TAM, and IL17RB^+^ILC2. Furthermore, combination with ICB revealed a synergistic antitumor effect [210]. Nevertheless, these data demonstrate the exploitation of the IL-33/ST2 axis as a promising target of cancer immunotherapy (Figure 3). Considering the dual function of IL-33, optimal targeting strategies should be developed to suppress the pro-tumorigenic effects while driving anti-tumor activities of IL-33 preferentially, depending on the specific environmental context. There are also notable considerations on the limitation of the clinical application of IL-33-mediated immunotherapy. The development of rational strategies to mitigate the dose-limiting toxicities is required to maximize the selective delivery of IL-33 to the site of action, the TME minimizing systemic exposure using various delivery platforms, such as immunocytokine fusions, oncolytic viral vectors, and nanoparticles.

## 6. Concluding Remarks

Tumor initiation and progression are tightly linked to the regulation of inflammatory cytokines. The summarized findings in this review show that IL-33 could be highly expressed in normal epithelium, suggesting its role in immune surveillance during infection, allergies, and tissue damage, etc. Along with tumor development, IL-33 is likely downregulated in epithelial cells but upregulated in the TME and serum despite the existing contradictory results. Nevertheless, the increased IL-33 expression in stromal components, such as CAF, creates a highly suppressive milieu for the immune response, thus contributing to the tumor progression and metastasis mainly by recruiting immune-suppressive TAM, MDSC, and T_reg_. Moreover, the low level of serum IL-33 might elicit systemic inflammation, leading to the mobilization and proliferation of BM hematopoietic progenitor cells that facilitate metastatic progression [211]. Apart from the differential expression level of IL-33 depending on physical locations, the involvement of other inflammatory cytokines also affects its function. Indeed, the presence of cytokines, such as IL-12 together with IL-33, promote type 1 anti-tumor immune responses. The majority of studies reveal that systemic and/or local production of IL-33 exerts a potent inhibitory effect on tumor growth and metastasis by enhancing both adaptive (i.e., effector Th1 and CD8^+^ T cells) and innate (i.e., NK cells, DCs, and eosinophils) immune components, and yields further synergy in combination with ICB. In contrast, IL-33, together with immune suppressive mediators and cytokines, such as TGF-β, in the TME, may potentiate further immune suppression at least in part through T_reg_, TAM, and MDSCs. The underlying mechanisms for the regulation of IL-33 expression in the TME remain still elusive. Considering that IL-33 exerts its function by binding to its receptor ST2 on target cells, the expression of ST2 and sST2 or the ratio of ST2/sST2 will be a critical regulatory step for directing pro- and anti-tumor activity by IL-33. The underlying mechanisms for the ST2/sST2-mediated modulation are still ill-defined and intensive investigations need to be performed. 

A clear understanding of the distinct roles of IL-33 in the epithelial, stromal, and immune cell compartment in TME is becoming increasingly important in developing more effective therapeutic approaches that include ICB, adoptive T cell therapy, oncolytic immunotherapy, and antibody-cytokine fusion therapy [212,213,214]. Notably, the intervention of the IL-33/ST2 axis for cancer therapy should be approached with caution, given IL-33 is a pleiotropic cytokine with the nature of a double-edged sword, namely pro- and anti-tumorigenic functions.

## Figures and Tables

**Table 1:** Pro- and anti-tumor effects of IL-33 in cancer.

Role	Mechanism of Action of IL-33/ST2	Cancer Type (H, Human) (M, Mouse)	References
Pro-tumorigenic	Immune cell-independent		
	-Promote tumor angiogenesis.	CRC (H/M)	[13]
	-Induce cancer cell stemness via activation of JNK or NF-kB pathway.	Breast (H/M) CRC (H/M)	[14,15]
	-Promote EMT and tumor cell proliferation, invasion, and metastasis by elevating MMP2/9 and other inflammatory cytokines (e.g., IL-6 and IL-17) and/or soluble mediators (e.g., COX2/PGE).	Breast (H) CCA (H/M) CRC (H/M) Lung (H/M)	[13,16,17,18,19,20,21]
	-Mediate CAF-induced tumor invasiveness and metastasis.	CRC (H/M) HNSCC (H) OSCC (H/M) Pancreatic (H/M)	[22,23,24,25]
	Immune cell-dependent		
	-Favor recruitment and inhibitory activities of immunosuppressive myeloid cells (e.g., TAM, MDSCs, and neutrophils), T_regs_, and ILC2s.	Breast (H/M) CRC (H/M)	[13,15,16,26,27,28]
Anti-tumorigenic	Immune cell-independent		
	-Inhibit cancer cell growth by downregulation of proteins involved in cellular proliferation and by upregulation of pro-apoptotic molecules.	Colon (H) Pancreatic (H)	[29,30]
	Immune cell-dependent		
	-Promote the MHC-I and IFN-γ-mediated immune surveillance.	Breast (M) Cervical (H) Colon (M) Lung (M) Prostate (H/M)	[31,32,33,34,35]
	-Inhibit tumorigenesis via modulation of B cell-produced IgA, IL-1α, and the microbiota.	CAC (M)	[36]
	-Inhibit tumor cell propagation and metastasis via the cooperation of Th1, CD8^+^ T cells, NK, DCs, and eosinophils.	AML (M) Breast (M) Lung (M) Lymphoma (H/M) Melanoma (M) Prostate (H/M)	[32,34,35,37,38,39,40,41,42]

AML, acute myeloid leukemia; CAC, Colitis-associated cancer; CAF, cancer-associated fibroblasts; CCA, cholangiocarcinoma; CRC, colorectal cancer; HNSCC, head and neck squamous cell carcinoma; NSCLC, non-small cell lung carcinoma; OSCC, oral squamous cell carcinoma.
